# Double Eyelid Tape Wear Affects Anterior Ocular Health among Young Adult Women with Single Eyelids

**DOI:** 10.3390/ijerph17217701

**Published:** 2020-10-22

**Authors:** Pui Theng Yong, Norlaili Arif, Sharanjeet Sharanjeet-Kaur, Mohd Izzuddin Hairol

**Affiliations:** 1 Centre for Community Health Studies, Faculty of Health Sciences, Universiti Kebangsaan Malaysia, Kuala Lumpur 50300, Malaysia; araley0424@gmail.com (P.T.Y.); norlailiarif@ukm.edu.my (N.A.); 2 Centre for Rehabilitation & Special Need Studies, Faculty of Health Sciences, Universiti Kebangsaan Malaysia, Kuala Lumpur 50300, Malaysia; sharanjeet@ukm.edu.my

**Keywords:** double eyelid tape, anterior ocular health, comfort level, single eyelids

## Abstract

Many East Asians apply double eyelid tape to create the double eyelid effect temporarily as a means of increasing their beauty. This study evaluated the effects of four-week wear of double eyelid tape on anterior ocular health in young adult women with single eyelids. Twenty-nine participants who met the inclusion criteria were recruited. The participants’ anterior ocular health was examined including blinking characteristics (blink pattern and blink rate), ocular surface health (presence of corneal abrasion, corneal staining, conjunctival staining, corneal curvatures, meibomian gland dysfunction), tear break up time, intraocular pressure, and subjective comfort level. Participants were required to apply the double eyelid tape for at least eight hours a day and five days a week for four weeks. The parameters were re-measured at the end of each week. There was a significant increase in conjunctival staining, corneal staining, and meibomian gland dysfunction, with a significant reduction in tear break-up time and intraocular pressure. By week 3, all participants had incomplete blinks. There was no significant change in symptoms and subjective comfort level reported. Therefore, patients and eye care practitioners should be aware of the potential implications of double eyelid tape wear on ocular health, with no significant change in subjective comfort.

## 1. Introduction

Facial structure and appearance can be distinguished between different ethnicities due to genetics [1,2]. People with Chinese ancestry can be easily recognized by the unique appearance of the eye as they have a narrower vertical palpebral fissure and an absence of a supra-tarsal fold compared to other races [1,3].

The appearances of the ‘East Asian eyelid’ can be categorized into three main categories: single eyelid, low eyelid crease, and double eyelid [1,4]. These can be differentiated through external observation: single eyelid has no apparent lid crease, low eyelid crease has a low-seated, nasal tapered crease with hidden fold, and double eyelid has a well-formed supra-tarsal crease. The prevalence of Asians born with double eyelid is 50% [5], while the prevalence of double eyelid among Malaysians with Malay and Chinese ethnicity is 100% and 70.1%, respectively [6].

Different ethnicities may have different views on standards of beauty [7]. Due to the influence of westernizing trends among eastern cultures, big and round eyes became an ideal image for Asian females [8,9]. Therefore, cosmetic products or surgical approaches are sought after by East Asians to obtain this ideal image [9,10,11].

Double eyelid ‘makeup’ is commonly used by Asians to create an artificial fold on the upper eyelid thus creating the double eyelid effect temporarily. It can be categorized into two main types: single-sided [12] and double-sided [13]. The second type uses double-sided eyelid tape or liquid glue, which creates a fold by attaching to the eyelid skin. This is better than surgical procedures, i.e., blepharoplasty, to achieve the double eyelid effect, as the tape can be removed, and the effect is not permanent. 

The use of conventional eye cosmetics, such as mascara, is known to cause ocular discomfort such as dry eyes [14,15] with various ocular complications including corneal trauma [16]. As ocular health and comfort are associated with one’s quality of life [17,18,19,20], the use of double eyelid tape raises questions as to whether it alters the user’s blinking pattern thus leading to lipid deficiency dry eye syndrome [21], or changes in corneal curvature and intraocular pressure (IOP) due to a possible compression from the artificially created eyelid fold. Although the adverse effects of conventional eye cosmetics have been reported [14,22,23], there are no published data on the effects of prolonged wear of double-sided eyelid tape on anterior ocular health and comfort.

Thus, the current study was conducted to determine: (1) blinking characteristics (blink pattern and blink rate), (2) ocular surface health (presence of corneal abrasion, corneal staining, conjunctival staining, corneal curvatures, Meibomian gland dysfunction), (3) tear break-up time, (4) intraocular pressure, and (5) subjective comfort level, all in young females with single eyelid, wearing double eyelid tape for four weeks.

## 2. Materials and Methods

### 2.1. Sample Size Calculation

An a priori power analysis was conducted with G*Power version 3.1.9.2 [24] using one-way repeated measures ANOVA (analysis of variance) (within factors). The calculated sample size of 26 participants gave the study a power of 0.91 with an effect size of 0.25 and a margin of error at 5%.

### 2.2. Study Location

All data collection was conducted at the Specialty Contact Lens Clinic housed within the Optometry and Vision Science Program, Faculty of Health Sciences, Universiti Kebangsaan, Malaysia between May and December 2019.

### 2.3. Participant Selection

Participants were females of Chinese ancestry, aged between 18–29 years old recruited via purposive sampling. Inclusion criteria included having single eyelids and having not used a double eyelid tape for the past six months.

All participants received written and oral information about the study and gave informed consent. A comprehensive survey was conducted to record participants’ ocular disease status and history, systemic disease status and history, allergen information and medication, and supplementary intake status. A slit lamp examination was conducted to determine the eyelid morphology and the signs of any anterior ocular disease. McMonnies’ Dry Eye Questionnaire was administered to screen potential participants with symptoms of dry eyes.

Participants were excluded if they had any ocular diseases, including dry eye, conjunctivitis, keratitis, keratoconjunctivitis, blepharitis, corneal degeneration, meibomian gland dysfunction (MGD), and ptosis. Those with known systemic diseases and under ocular and systemic medications were also excluded. Pregnant participants were excluded from this study, as well as those wearing rigid gas permeable (RGP) and soft contact lenses.

The conduct of this research followed the tenets of the Declaration of Helsinki. Research ethics approval was granted by the Research Ethics Committee, Universiti Kebangsaan, Malaysia (UKM PPI/111/8/JEP-2019-310).

### 2.4. Study Procedures

All study procedures were conducted by a graduate research student with three-years clinical experience as a registered optometrist. Week 0 was designated as the time when all variables were measured for the first time, before wearing the double eyelid tape. First, all participants responded to the Ocular Surface Disease Index© (OSDI©) questionnaire. Next, horizontal and vertical corneal curvatures were measured using the TOMEY TMS-4N (Cecil Swyers, Phoenix, AZ, USA) corneal topographer. A Righton MW50D video-recording slit lamp (Right Mfg Co Ltd., Japan) was used to assess the anterior ocular structures. The participants were not informed that their blink pattern and blink rate were observed to avoid bias that could have affected their blinking characteristics.

Fluo Strip fluorescein stain was applied to the participant’s right eye and tear film was observed under cobalt blue light with medium magnification. Participants were instructed and encouraged to blink normally. Blink pattern and blink rate were observed and video-recorded for 30 s. Blink pattern was defined as complete when there was a full blink and incomplete if the blink was partial. Blink rate was calculated as the number of blinks made (irrespective whether complete or incomplete) per minute. Tear break-up time (TBUT) was measured by asking the participants to force close the eyes, followed by opening their eyes without blinking. The time when the fluorescein-stained tear spread on the corneal surface started to break was noted, which was also determined using video recording. The presence of corneal abrasion was observed as either present or absent. This was followed by gradings of corneal staining, conjunctival staining, and meibomian gland dysfunction. These were determined using the Efron Grading Scale as guidance to grade the condition in five levels: normal (0), trace (1), mild (2), moderate (3), and severe (4) in 0.5 unit increments. Intraocular pressure was measured using the Goldmann applanation tonometer (HAGG-STREIT AT 900 model T applanation tonometer, Hagg-streit AG, Switzerland). Before the measurement of intraocular pressure (IOP), topical anesthetic (Alcaine 0.5%) was instilled followed by punctal occlusion and corneal sensitivity test using the cotton tip of a cotton bud.

**Figure 1:**
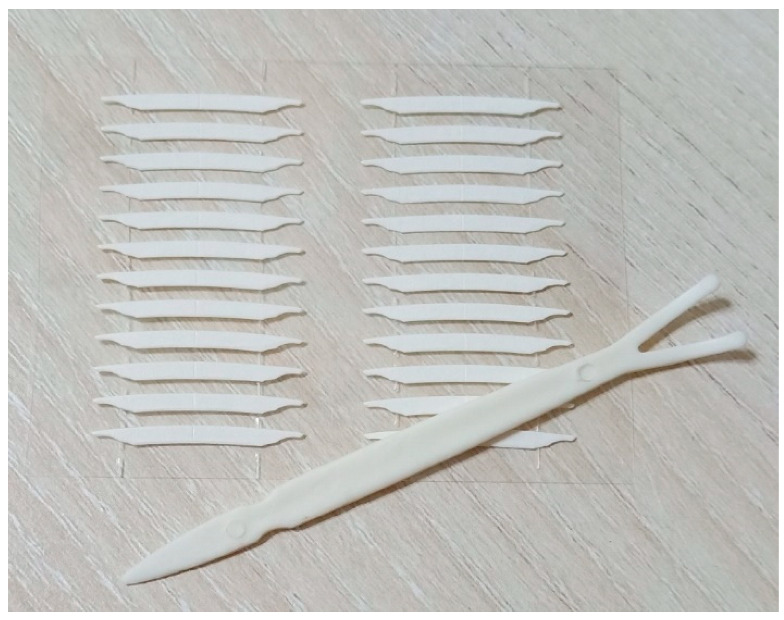
A set of double eyelid tape and the y-shaped application tool (Original Equipment Manufacturer (OEM), Qingdao, China).

Then, participants were supplied with a set of double eyelid tapes and its application tool (Original Equipment Manufacturer (OEM), Qingdao, China), shown in Figure 1. Participants were taught to use the pointed end of the applicator to take out the tape from its packaging, then apply it around 4 to 6 mm above the upper eyelid margin. The white, non-sticky outer later sheet was then removed before the Y-shaped applicator was used to gently push the upper eyelid upwards to create an eyelid fold. This was followed by a gentle pinch on the newly created eyelid fold to ensure the adhesion of the double eyelid tape. Participants were also taught to clean their eyelids using a wet cotton pad, gently with a circular motion to remove excess dirt or oil on the eyelid, before applying the double eyelid tape to enhance its adhesion, and to repeat the step in order to loosen the tape adhesion for its removal. Participants wore the double eyelid tape for no less than five days in a week without other eye makeup and contact lens wear and for at least eight hours per day.

All ocular parameters of interest were measured every seven days for the next four weeks. However, the OSDI questionnaire was only administered in Week 0 and Week 4. The measurements made in Week 1 to Week 4 were performed while the participants were wearing the double eyelid tape. All gradings were carried out by author PTY. The video recordings were also evaluated by author NA to minimize bias in the grading process.

### 2.5. Statistical Analysis

Raw data were sorted in Microsoft Excel 2016 (Redmond, WA, USA), then analyzed using IBM SPSS Statistics version 25 (IBM Corp., Armonk, NY, USA). Cochran’s Q test was used to test the changes in proportion of participants’ blink patterns and the presence of corneal abrasion after the intervention of double eyelid tape wear. Differences in means between the weekly measurements for corneal curvature, TBUT, and IOP were analyzed using one-way repeated-measures analysis of variance (ANOVA) with Hyunh-Feldt corrections, while MGD were analyzed by a sphericity assumed test of one-way repeated measures analysis of variance (ANOVA). Conjunctival staining, corneal staining, and blink rate measured across the four weeks were analyzed using the Friedman test. OSDI© (Allergan plc, Irvine, CA, USA) scores were compared before and after the double eyelid tape intervention and analyzed using the Wilcoxon signed-rank test. For all tests, significance was assumed to be *p* < 0.05.

## 3. Results

Although sample size calculation showed that 26 participants were needed, we managed to recruit 29 young adults to participate in this study. All participants were female Malaysians with Chinese ancestry and had single eyelids. The mean age of the participants was 21.83 ± 1.97 years, ranging from 19–28 years. The McMonnies Dry Eye Questionnaire score, measured during participant recruitment, ranged between 1–9 (mean: 5.00 ± 2.17).

### 3.1. Blink Characteristics

**Figure 2:**
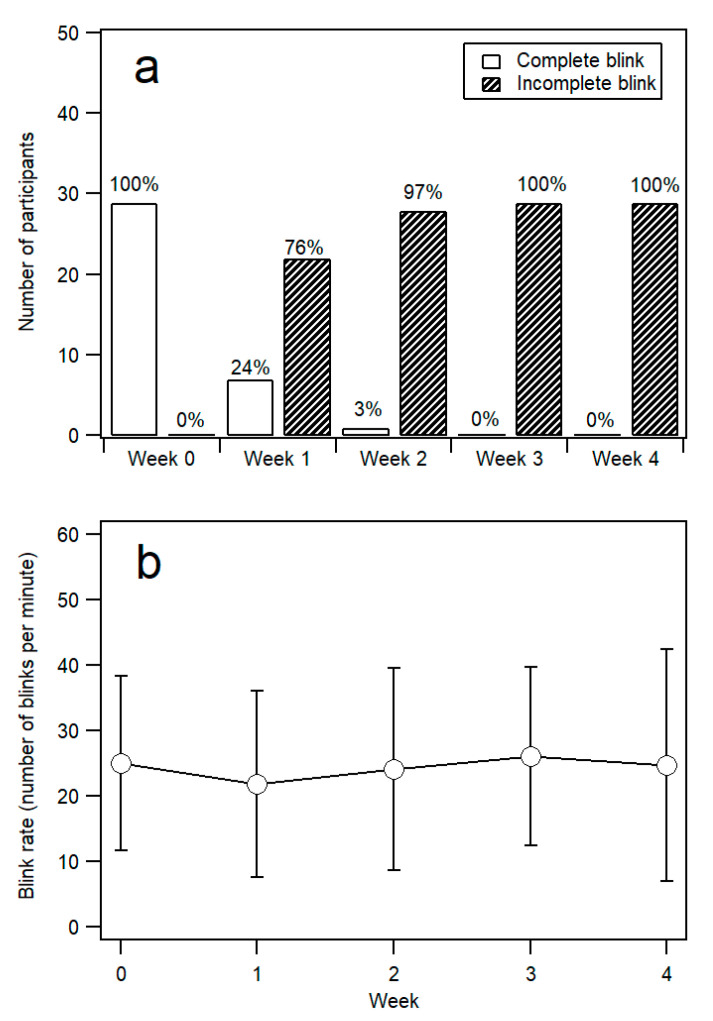
(**a**) Blink pattern (completeness of blink) and (**b**) mean blink rate (number of blinks per minute) from Week 0 (before eyelid tape wear) to Week 4. Error bars represent ±1 standard deviatiom (SD).

Figure 2a compares the blink pattern (completeness of blink) from Week 0 through to Week 4. Before double eyelid tape wear, all participants had complete blink. By Week 1, only seven participants (24%) had complete blink. By Week 2, there was only one participant (3%) who still showed complete blink. By Week 3, none of the participants showed complete blink, i.e., all 29 participants demonstrated incomplete blink. The change in proportion of blinking pattern was statistically significant (Cochran’s Q (4, *N* = 29) = 94.95, *p* < 0.001). Pairwise comparison with McNemar test and Bonferroni correction showed that the differences between Week 0–Week 1 and Week 0–Week 4 were highly significant (all *p* < 0.001).

Figure 2b compares the blink rate from Week 0 to Week 4. In Week 0, before the application of double eyelid tape, the blink rate was 24.97 ± 13.38 blinks per minute (bpm). Throughout the assessment weeks, there was no obvious trend in the change of the blink rate of the participants. A Friedman two-way ANOVA indicated that there was no significant difference in rankings of blink rate during the five visits from Week 0 to Week 4, [χ^2^_F_ = 3.71 (corrected for ties), *df* = 4, *N*–Ties = 29, *p* = 0.447].

### 3.2. Ocular Surface Health

#### 3.2.1. Corneal Abrasion

Corneal abrasion was absent for all participants on Week 0. It was present in two participants in Week 1, three participants in Week 2 and Week 3, and one participant in Week 4. The differences between the related proportions of corneal abrasion was not statistically significant, [Cochran’s Q (4, *N* = 29) = 5.67, *p* = 0.225].

#### 3.2.2. Corneal and Conjunctival Staining

**Figure 3:**
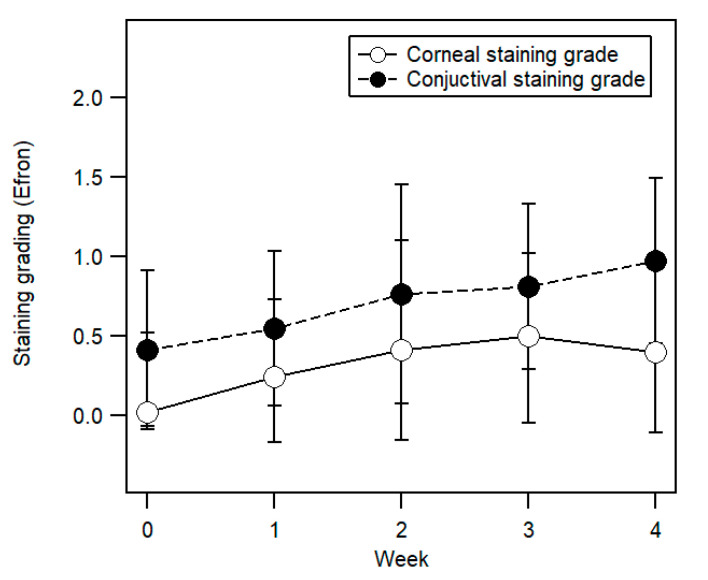
Mean of corneal staining grade (open circles) and mean of conjunctival staining grade (closed circles) from Week 0 (before eyelid tape wear) to Week 4. Error bars represent ±1 SD.

The grading of corneal staining showed a general increasing trend from Week 0 to Week 4, as shown in Figure 3 (open circles). In Week 0, the mean corneal staining grade was 0.02 ± 0.09 (95% confidence interval, CI (−0.02, 0.00)), increasing to a peak grading of 0.50 ± 0.55 (95% CI (−0.30, 0.80)) in Week 3, before decreasing to a mean grade of 0.40 ± 0.51 (95% CI (0.21, 0.61)) in Week 4. A Friedman two-way ANOVA indicated that rankings of corneal staining grading varied significantly across the five visits from Week 0 to Week 4 (χ^2^_F_ = 21.69 (corrected for ties), *df* = 4, *N*–Ties = 29, *p* < 0.001). Corneal staining grading in Week 2, Week 3, and Week 4 was significantly higher than that for Week 0 (Wilcoxon Signed Rank pairwise comparisons, all *p* < 0.05).

Conjunctival staining grade also showed a similar increasing trend from Week 0 to Week 4 (Figure 3, closed circles). In Week 0, the mean grade was 0.41 ± 0.50 (95% CI (0.23, 0.60)) and reached a peak grading of 0.97 ± 0.52 (95% CI (0.78, 1.15)) in Week 4. The rankings of conjunctival staining varied significantly across the five visits (Friedman two-way ANOVA, (χ^2^_F_ = 20.93 (corrected for ties), *df* = 4, *N*–Ties = 29, *p* < 0.001)). Conjunctival staining grading in Week 2, Week 3, and Week 4 was significantly higher than that for Week 0 (Wilcoxon Signed Rank pairwise comparisons, all *p* < 0.05).

#### 3.2.3. Horizontal and Vertical Corneal Curvature

Mean horizontal corneal curvature was similar throughout the study period (Week 0: 43.35 ± 1.45 D (95% CI (42.81, 43.88)) and Week 4: 43.28 ± 1.40 D (95% CI (42.77, 43.79)) and any differences were not statistically significant (F(2.15, 60.21) = 2.27, *p* = 0.12, partial *η^2^* = 0.08). Mean vertical corneal curvature also had similar trend and differences throughout Week 0 to Week 4 was also not statistically significant (F(1.63, 45.72) = 0.552, *p* = 0.55, partial *η^2^* = 0.02).

#### 3.2.4. Meibomian Gland Dysfunction

**Figure 4:**
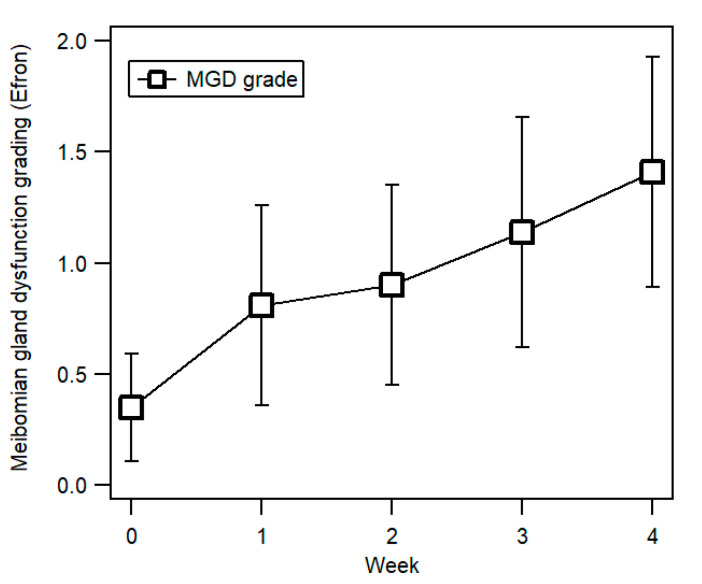
Meibomian gland dysfunction grade (open circles) from Week 0 (before double eyelid tape wear) to Week 4. Error bars represent ±1 SD.

The grading for meibomian gland dysfunction (MGD) showed an increasing trend from Week 0 to Week 4, as evident in Figure 4. In Week 0, the mean MGD grade was 0.35 ± 0.24 (95% CI (0.26, 0.43)), reaching a peak grade of 1.41 ± 0.52 (95% CI (1.22, 1.60)) in Week 4. A Friedman two-way ANOVA indicated that rankings of MGD grading increased significantly across the five visits from Week 0 to Week 4, (χ^2^_F_ = 72.76 (corrected for ties), *df* = 4, *N*–Ties = 29, *p* < 0.001). Follow-up pairwise comparisons with the Wilcoxon Signed Rank test showed that MGD grading between Week 0 and the other four weeks varied significantly (all *p* < 0.001).

### 3.3. Tear Break-Up Time (TBUT)

**Figure 5:**
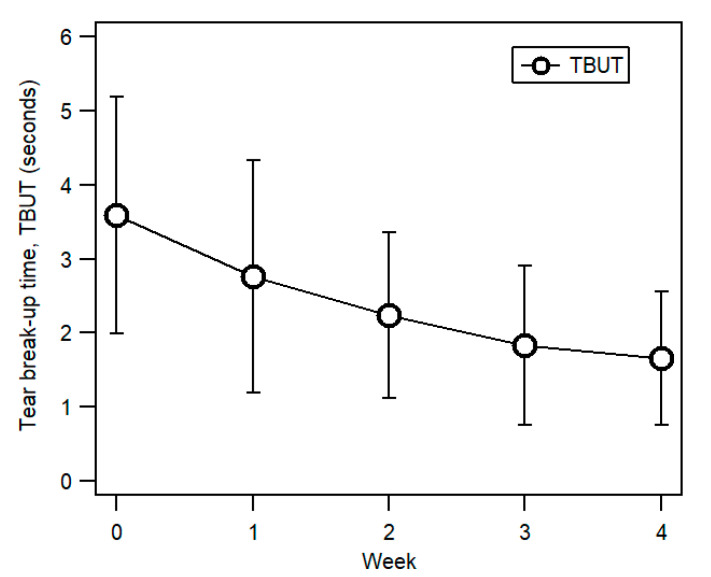
Mean of tear break-up time (TBUT) measured in seconds from Week 0 (before eyelid tape wear) to Week 4. Error bars represent ±1 SD.

TBUT decreased throughout the study period (Figure 5), with a mean of 3.59 ± 1.60 s (95% CI (3.01, 4.17)) in Week 0, reaching a minimum of 1.66 ± 0.90 s (95% CI (1.33, 1.98)) in Week 4. The decrease in TBUT was statistically significant, (F(3, 83.90) = 20.24, *p* < 0.001, partial *η^2^* = 0.42) with pairwise comparison showing TBUT in Week 2, Week 3 and Week 4 to be significantly lower than in Week 0 (all *p* < 0.001).

### 3.4. Intraocular Pressure (IOP)

**Figure 6:**
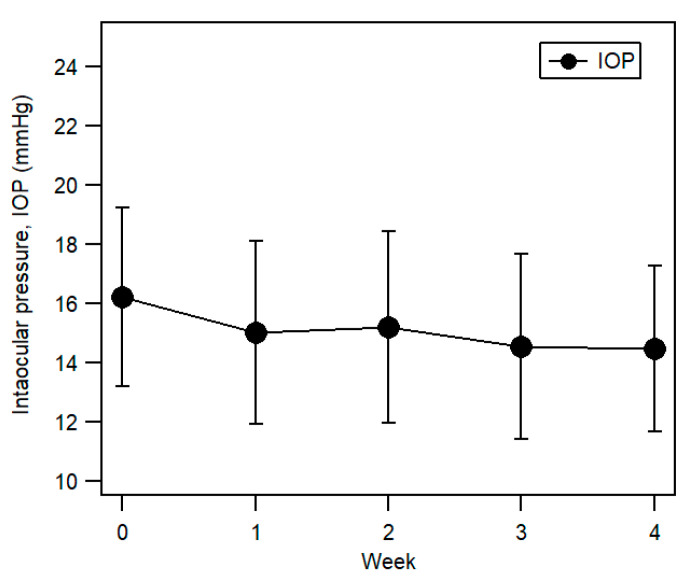
Mean intraocular pressure (IOP) from Week 0 (before eyelid tape wear) to Week 4. Error bars represent ±1 SD.

There was a decreasing trend in IOP (Figure 6) between Week 0 (16.21 ± 3.03 mmHg (95% CI (15.10, 17.31)) and Week 4 (14.45 ± 2.81 mmHg (95% CI (13.43, 15.47)) and was statistically significant (F (3.45, 96.46) = 4.41, *p* < 0.01, partial *η^2^* = 0.14). Pairwise comparisons further revealed that the IOP on Week 1, Week 3, and Week 4 were significantly lower than Week 0 (all *p* < 0.05).

### 3.5. Ocular Surface Disease Index (OSDI) Score

The mean of OSDI score was higher in Week 4 (11.12 ± 10.90 (95% CI (7.00, 16.98)) compared to Week 0 (9.98 ± 8.18 (95% CI (7.16, 18.28)). However, the score difference before and after the application of double eyelid tape was not statistically significant (*T* = 168.50, *z* = −0.79 (corrected for ties), *N*–Ties = 29, *p* = 0.432, two-tailed).

Table 1 summarizes the findings for all the parameters measured from Week 0 to Week 4.

## 4. Discussion

The study was conducted to investigate the effects of four–week double-sided eyelid tape wear on the anterior ocular structures and ocular health in young adult women with single eyelids. Participants showed a significant increase in the occurrence of incomplete blinking after four weeks of use. This could be due to restricted movements of the upper eyelid induced by double-sided eyelid tape wear. A study reported that the use of double eyelid glue led to the obstruction of eyelid closure, increasing tear evaporation rate, and adversely affecting meibomian gland secretion [25].

Incomplete blinking, therefore, might be related with other findings reported in this study, as we found a statistically significant increase in meibomian gland dysfunction (MGD) grading, conjunctival staining, and corneal staining. The inability to blink completely for more than one-week might contribute to the occurrence of MGD [21]. Incomplete blinking has also been associated with increased corneal staining [26] affecting ocular surface health due to inadequate lipid distribution and continuous exposure of the inferior ocular surface [27]. This also leads to other complications such as superficial punctate keratopathy [28], corneal staining, an increase in dry eye symptoms [29] and fast evaporation of tears [21].

Although the staining grading increased by only about one level (from 0 to between 0.5 and 1.5), the effect was statistically significant. However, these increases were from ‘normal’ to ‘mild’ based on the Efron Grading Scale classification. As the double eyelid tape was removable, it might take longer than four weeks of wear for these clinical changes to become more clinically significant. Nevertheless, we have showed that these changes were clearly evident when using the tape during the day for four weeks. Furthermore, the study participants were not wearing other eye cosmetics. The grading could be higher, i.e., worse, if double eyelid tape was used with other eye cosmetics, as the cosmetics themselves have been reported to have adverse effects on the anterior eye health. For example, eye cosmetics such as mascara and eyeliner could cause lacrimal outflow blockage, tear film instability, and meibomian gland obstruction [25,30,31].

Double eyelid tape wear also resulted in a significant reduction in tear break-up time (TBUT). Incomplete blinking is known to lead to inadequate lipid distribution due to continuous exposure of the inferior ocular surface that also increases tear evaporation [27]. Indeed, ocular surface changes can occur due to the fast evaporation of tears due to incomplete blinking and MGD. The results of this study showed that double eyelid tape wear led to incomplete blinks, thus contributing to ocular surface dryness. Continuous drying out of the ocular surface due to reduced TBUT causes the formation of dry spots on the ocular surface, mostly found inferiorly as a result of consequent exposure of the inferior part of the ocular surface, due to uneven lipid distribution [21]. Participants in this study had low TBUT values at baseline (3.59 ± 1.60 s). Various studies comparing TBUTs for populations of different ethnicities have also shown that East Asians have generally shorter TBUT [32,33,34], with up to 80% asymptomatic Asians also having TBUT below the threshold for dry eye diagnosis [34,35]. Several studies conducted in Malaysia have also shown that young adults in the country had low TBUT values without dry eye symptoms: 5.69 ± 1.61 s [20], 5.51 ± 1.44 s [36], and 3.00 ± 2.61 s [37]. The TBUT findings of [37] were most similar to ours, where participants in both studies were all young females. These low TBUT values could be attributed to an earlier report that females had faster breakup times than males [38]. Besides, the decrease in TBUT of the participants in this study throughout the four-week study period could also be due to incomplete blinking, which is associated with poorer tear film stability [35]. The combination of these factors may have resulted in rather low TBUT values at baseline for our participants.

In this study, double eyelid tape wear did not cause significant changes in both horizontal and vertical corneal curvatures. The amount of compression caused by the application and wear of double eyelid tape can be assumed to be inconsequential in its effect on the corneal curvature of the participants in this study. This is expected, as even long-term soft contact lens wear, which makes direct contact with the cornea, produces only very small changes in corneal curvature [39,40,41,42]. Double eyelid tape wear also did not cause a significant change in the presence of corneal abrasion. Several researchers have reported cases of mechanical corneal abrasion due to cosmetics appliances, such as mascara applicators and eyelashes curlers [43,44] and contact lens wear [45]. In this study, superficial corneal abrasion was observed in several participants. It was typically resolved within one week and was not observed on the next visit. However, superficial corneal abrasion in two participants was present for three consecutive visits, likely due to an accidental scratch on the ocular surface caused by the double eyelid tape applicator. Corneal abrasion could also occur due to itchiness induced by dry eye [46]. However, mechanical corneal abrasion could be avoided with careful practice while using the double eyelid tape applicator.

There was a statistically significant reduction in IOP throughout the study period, which was unexpected considering extrinsic compression on the globe, such as eye rubbing (and presumably, the process of applying the double eyelid tape) could cause alteration of intraocular pressure [47]. At the beginning of the current study, measurements of IOP might have been higher as a majority of our participants had never gone through IOP measurement using the Goldmann applanation tonometer. It could be assumed that relatively higher IOP obtained from the first two visits was overestimated, as anxiety or stress could induce higher IOP [48].

The double eyelid effect can also be achieved surgically with full-incision blepharoplasty. The procedure may alter the patients’ tear film dynamics and increase dry eye symptoms, although the effects are resolved within three months after the surgery [49,50] Some patients may prefer double eyelid tape to blepharoplasty due to the former being of non-invasive nature, of lower cost, and irreversible. However, both patients and practitioners must be aware of the effect of the tape on the anterior ocular health especially when it is worn for a relatively extended period (that is, four weeks in this study). Although there were significant changes in some of the ocular health parameters, there were no significant differences in OSDI scores. As OSDI is a tool to assess the severity of dry eyes [51], the changes in parameters such as TBUT and MGD within the four-week period might not have been sufficient to cause any significant changes in its scores. Although not statistically significant, the increase in OSDI scores at Week 4 may indicate a possible public health concern with regards to double eyelid tape wear. Indeed, an increase in OSDI score, even when not significant statistically, may be real and clinically meaningful [52]. Longer duration of tape wear may lead to more prevalent reported symptoms, which should be addressed in future work.

In this study, the bias in gradings of clinical signs were controlled and reduced using video recording of blinking characteristics. The participants were also not informed that their blink characteristics were being observed to avoid these parameters from being consciously altered. Author NA was also tasked to grade some of the ocular findings in which all recordings were not associated with any participants. However, some limitations remained. Although gradings using the Efron scale between authors PTY and NA agreed with each other, they were done on selected randomized samples. It is possible that some expectation bias, although minimized, might be present.

## 5. Conclusions

Four-week period of double eyelid tape wear had a significant effect on ocular surface health, namely, an increase in conjunctival staining, corneal staining, corneal abrasion, and meibomian gland dysfunction grading, with a significant reduction in tear break-up time and intraocular pressure. However, there was no change in subjective comfort reported by the participants. Longer duration of double eyelid tape wear can potentially result in signs and symptoms that are clinically significant. Patients and eye care practitioners should be aware of the potential implications of prolonged double eyelid tape wear on ocular health.

## Figures and Tables

**Table 1:** The Mean ± Standard Deviation (SD), 95% Confidence Interval (CI) and *p* Values of Parameters Measured in Weeks 0, 1, 2, 3, and 4.

Parameter	Week 0		Week 1		Week 2		Week 3		Week 4		*p* Value
	Mean ± SD	95% CI	Mean ± SD	95% CI	Mean ± SD	95% CI	Mean ± SD	95% CI	Mean ± SD	95% CI	
**Blink rate (bpm)**	24.97 ± 13.38	20.1, 29.84	21.79 ± 14.26	16.6, 26.98	24.07 ± 15.46	18.44, 29.70	26.00 ± 13.67	21.02, 30.98	24.69 ± 17.69	18.25, 31.13	0.45
**Corneal staining grade**	0.02 ± 0.09	−0.02, 0.00	0.24 ± 0.41	0.09, 0.33	0.41 ± 0.57	0.21, 0.62	0.50 ± 0.55	0.30, 0.80	0.40 ± 0.51	0.21, 0.61	<0.001
**Conjunctival staining grade**	0.41 ± 0.50	0.23, 0.60	0.55 ± 0.49	0.37, 0.73	0.76 ± 0.69	0.51, 1.01	0.81 ± 0.52	0.62, 1.00	0.97 ± 0.52	0.78, 1.15	<0.001
**Horizontal corneal curvature (D)**	43.35 ± 1.45	42.82, 43.88	43.28 ± 1.39	42.77, 43.78	43.28 ± 1.40	42.77, 43.78	43.32 ± 1.44	42.79, 43.84	43.28 ± 1.40	42.77, 43.79	0.12
**Vertical corneal curvature (D)**	44.88 ± 1.52	44.33, 45.44	44.91 ± 1.49	44.36, 45.45	44.81 ± 1.37	44.31, 45.31	44.83 ± 1.55	44.27, 45.39	44.84 ± 1.49	44.30, 45.38	0.55
**Meibomian gland dysfunction** **(MGD) grade**	0.35 ± 0.24	0.26, 0.43	0.81 ± 0.45	0.65, 0.97	0.90 ± 0.45	0.73, 1.06	1.14 ± 0.52	0.95, 1.33	1.41 ± 0.52	1.22, 1.60	<0.001
**Tear break-up time (TBUT) (s)**	3.59 ± 1.60	3.01, 4.17	2.76 ± 1.57	2.19, 3.33	2.24 ± 1.12	1.83, 2.65	1.83 + 1.07	1.44, 2.22	1.66 ± 0.90	1.33, 1.98	<0.001
**Intraocular pressure (IOP) (mmHg)**	16.21 ± 3.03	15.10, 17.31	15.03 + 3.07	13.92, 16.15	15.21 ± 3.25	14.03, 16.39	14.55 ± 3.11	13.42, 15.68	14.45 ± 2.81	13.43, 15.47	<0.01
**Ocular Surface Disease Index (OSDI) score**	9.98 ± 8.18	7.00, 16.98	-	-	-	-	-	-	11.12 ± 10.90	7.16, 18.28	0.43
